# Comparative analysis of different macromolecular crowding agents in human tenocyte cultures

**DOI:** 10.1016/j.bbiosy.2026.100127

**Published:** 2026-01-10

**Authors:** Andrea Rossoni, Giovanni Lauretta, Stephen Kearns, Dimitrios I. Zeugolis

**Affiliations:** a Regenerative, Modular & Developmental Engineering Laboratory (REMODEL), Charles Institute of Dermatology, Conway Institute of Biomolecular & Biomedical Research and School of Mechanical & Materials Engineering, University College Dublin (UCD), Dublin, Ireland; b Bon Secours Hospital, Merlin Park Regional Hospital, University Hospital Galway and University of Galway, Galway, Ireland

**Keywords:** Extracellular matrix, Macromolecular crowding, Tendon engineering, Tendon cells

## Abstract

Tendon engineered substitutes remain clinically unavailable, largely due to the need for prolonged culture periods to allow for sufficient extracellular matrix deposition. Although macromolecular crowding allows for accelerated extracellular matrix deposition, the optimal macromolecular crowding agent in human tenocyte cultures is still unknown. Here, we measured the physicochemical properties of six macromolecules (κλ carrageenan, λ carrageenan, polysucrose, polyacrylic acid, hyaluronic acid and polyvinylpyrrolidone) and assessed their effect in human tenocyte basic function, collagen type I deposition and gene expression. The κλ carrageenan exhibited the highest polydispersity index. None of the macromolecules impaired cell viability and metabolic activity. The κλ carrageenan and the λ carrageenan significantly enhanced collagen type I deposition. A macromolecular crowding agent dependent gene expression was observed, with the κλ carrageenan appearing to induce a more mature phenotype. This study provides a framework for selecting an appropriate macromolecular crowding agent to enhance and accelerate extracellular matrix deposition, whilst maintaining acceptable cell function.

## Highlights

• κλ CR and λ CR induced the highest collagen type I deposition.
• Polydispersity index had the highest correlation to collagen type I deposition.
• Gene expression analysis revealed a MMC agent-dependent cell response.
• This work advocates the use of κλ CR and λ CR in tendon engineering.

## Graphical abstract

Identification of optimal macromolecular crowding agent in human tenocyte cultures.

**Figure:**
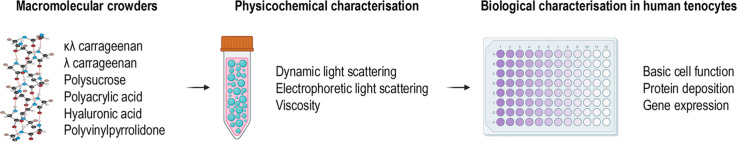

## 1 Introduction

Tendon injuries are a major clinical and socioeconomic burden. They are the fourth leading cause of work absenteeism in the U.S [1] and the most common cause of shoulder pain and disability worldwide, especially in the ageing population [2]. Globally, 6.4-7.1 million cases occur annually, with €25.46-28.49 billion associated healthcare costs [3]. Current treatments rarely restore full tendon function and are linked to several drawbacks. For example, physical therapy is insufficient for critically damaged tendons and lacks robust clinical evidence [4]. Corticosteroid injections are associated with various local and systemic side effects [5,6]. Platelet rich plasma injections have yielded highly heterogeneous outcomes largely due to differences in platelet rich plasma’s preparation, patient’s pathology and study’s design [7]. Tissue grafting approaches may be accompanied by rejection, disease transmission and donor-site morbidity [8,9]. Surgical repairs carry high re-tear rates and lead to adhesions that impair mobility [10]. Unfortunately, post-operation, many patients experience chronic pain and reduced load-bearing capacity [11]. These limitations resulted in the concept of tendon bioengineering that aims to develop living tendon substitutes to restore structure and function of injured tendons [[12], [13], [14]].

Clinical studies using autologous human tenocyte (hTC) implantation have demonstrated safety and modest improvements in pain and mobility over short and long-term follow up [15,16]. Autologous hTC transplantation has even allowed elite athletes to return to full training and competition [17,18] and is now considered as a safe and valuable option in the management of chronic tendinopathies [19]. Despite the positive patient therapeutic outcomes, the major challenge in autologous hTC transplantation is the functionality of the cells [20,21] after the extensive *in vitro* expansion required to generate the high cell numbers required (e.g. 4 weeks of culture are required to transplant 4,000,000-10,000,000 [22]).

An alternative strategy involves the development of tendon engineered constructs by combining cells, scaffolds, trophic molecules and appropriate microenvironmental signals [[23], [24], [25], [26], [27]]. Interestingly, although numerous studies have demonstrated positive therapeutic outcomes of non-human animal TCs [[28], [29], [30]] and human [[31], [32], [33]] and non-human animal [[34], [35], [36]] other cell populations, only one study has assessed hTC tissue engineered products in preclinical setting [37]. We attribute this to the plausible for non-human animal TC or other cell populations and human other cell populations but unlikely for hTC requirements. For example, three dimensional engineered tendons have been developed *in vitro* after 6 and 10 weeks of constant strain and no strain, respectively, using 25,000,000 avian TCs at passage 2 grown on polyglycolic acid unwoven fibres [38]. The reader should note that the development of a mechanically stable tissue engineered blood vessel based on human skin fibroblasts and saphenous vein endothelial cells required 28 weeks [39]. It has been argued that such prolonged culture periods are required due to the slow rate of extracellular matrix (ECM) accumulation *in vitro* [40,41].

Macromolecular crowding (MMC), a biophysical phenomenon that accelerates biological processes by reducing molecular diffusion and promoting molecular association [42,43], has been shown to enhance and accelerate ECM deposition in both permanently differentiated [[44], [45], [46], [47]] and multipotent [[48], [49], [50], [51]] cell cultures. MMC operates through the excluded volume effect theory, according to which two molecules cannot occupy the same space at the same time [[52], [53], [54]]. The physicochemical parameters, such hydrodynamic radius [55], polydispersity [56], charge [57] and viscosity [58], of the MMC agents have been shown to differentially affect volume exclusion / molecular diffusion and therefore the kinetics of biochemical and biological reactions. Further, in eukaryotic cell cultures, a MMC agent dependent cell behaviour has been observed. For example, polysucrose has been shown to enhance collagen type I deposition in human dermal fibroblasts [47] and human corneal fibroblasts [59], but to have a small effect in hTC cultures [60]. Further polysucrose has been shown to promote adipogenesis in human bone marrow mesenchymal stromal cell cultures [61], whilst carrageenan has been shown to promote osteogenic and chondrogenic commitment of human bone marrow mesenchymal stromal cells [62]. These findings clearly indicate that for a specific cell type the appropriate MMC agent should be identified.

Considering the above, it is imperative to develop tendon engineered medicines after short culture periods (to avoid issues associated not only with hTC phenotypic drift, but also with the manufacturing of prohibitively expensive for healthcare providers tendon engineered medicines) with the highest amount of tendon-specific ECM (to ensure not only fully functional tendon engineered medicines, but also hTC protection and localisation at the transplanted side). Bearing in mind the MMC agent dependent cell response and that hTCs have been shown to exhibit microenvironment-specific response (i.e. rigid substrates have been shown to upregulate osteogenic and chondrogenic genes [63]) that may lead to heterotopic ossification [64] or chondral metaplasia [65], it is crucial to identify the optimal MMC agent in hTC cultures. With these in mind, herein we first assessed the physicochemical properties (hydrodynamic radius, polydispersity, charge and viscosity) of the most commonly used MMC agents that have shown efficiency and efficacy in eukaryotic cell culture, namely κλ carrageenan (κλ CR) [66], λ carrageenan (λ CR) [67], polysucrose (PS) [68], polyacrylic acid (PAA) [69], hyaluronic acid (HA) [70] and polyvinylpyrrolidone (PVP) [71]. We then evaluated the effect of these MMC agents on hTC basic function, protein deposition and gene expression.

## 2 Materials and methods

### 2.1 Materials

κλ CR, PS (70 kDa and 400 kDa), PAA (4 MDa) and PVP (360 kDa) were sourced from Sigma Aldrich (Ireland). λ CR (Viscarin® PH-109) was bought from FMC Corporation (United States). The two HA molecules (60 kDa and 100 kDa) were obtained from Lifecore Biomedical (United States). Tissue culture supplies were purchased from Sarstedt (Germany). All other chemicals, reagents, cell culture consumables, laboratory consumables, etc. were purchased from Sigma Aldrich (Ireland), unless otherwise stated.

### 2.2 Physicochemical properties analysis

The hydrodynamic radius, polydispersity index (PDI), zeta potential and viscosity of each MMC agent in Hank’s Balanced Salt Solution (HBSS) were assessed at a literature-informed concentration (50 μg/ml κλ CR [66]; 10 μg/ml λ CR [67]; a cocktail of 37.5 mg/ml PS 70 kDa and 25 mg/ml PS 400 kDa [68]; 1 mg/ml PAA [69]; a cocktail of 10 mg/ml HA 60 kDa and 10 mg/ml HA 100 kDa [72]; and 25 mg/ml PVP). A Litesizer 500 (Anton Paar, Austria) system was used for dynamic (DLS) and electrophoretic (ELS) light scattering analyses. The samples were equilibrated at 37°C prior to analysis. For DLS, measurements (hydrodynamic radius and PDI) were performed using disposable polystyrene cuvettes (Sarstedt, Germany) and runs were recorded at a scattering angle of 175 ° (backscatter). For ELS, zeta potential measurements were performed with reusable omega cuvettes (Anton Paar, Austria) and runs were recorded at a scattering angle of 15 ° (forward scattering). The viscosity was determined using an MCR 302 rheometer equipped with a 50 mm cone-plate configuration (both Anton Paar, Austria). The samples were equilibrated at 37°C for 5 min and kept constant for the whole analysis. A pre-shear at 10 Hz for 1 min was applied followed by a shear rate ramp from 0.0001 Hz to 1000 Hz for 12.5 min. The viscosity (μ) of each MMC agent solution was determined by fitting data points to a linear equation and calculating the slope of the shear stress (τ) versus shear rate $\left(\dot{\gamma }\right)$ relationship.

### 2.3 Cell isolation and culture

The cells were isolated from the Achilles tendon of a healthy 25-year-old female donor (licence CA 1046 of Dimitrios Zeugolis, University of Galway, Galway, Ireland), using the established migration protocol [73]. The cells were expanded to passage 3 in Dulbecco’s Modified Eagle Medium with 4.5 g/l D-glucose, GlutaMAX™-1, pyruvate (DMEM, ThermoFisher Scientific, Ireland), 10 % foetal bovine serum (FBS, Gibco, United Kingdom) and 1 % penicillin-streptomycin (PS, Gibco, United Kingdom). At passage 4, the cells were seeded at 25,000 cells/cm² and allowed to attach for 24 h. The culture medium was then replaced with fresh medium containing either DMEM, 10 % FBS, 1 % P/S and 100 μM L-ascorbic acid phosphate (-MMC) or DMEM, 10 % FBS, 1 % P/S, 100 μM L-ascorbic acid phosphate and each of the MMC agents. Each media was replaced with respective media every other day and read outs were obtained at days 4, 6 and 8.

### 2.4 Basic cell function analysis

Cell morphology was assessed at each time point with an Olympus CKX 53 (Olympus Corporation, Japan) inverted microscope and the captured images were processed using ImageJ (version 2.16.0, National Institute of Health, United States).

Cell metabolic activity was assessed by alamarBlue® assay (Invitrogen, ThermoFisher Scientific, Ireland) according to manufacturer’s instructions. Cells were washed with HBSS and incubated with 10 % alamarBlue® solution in HBSS for 3 h at 37°C and 5 % CO₂. Absorbance was measured at 570 and 600 nm using a FLUOstar® Omega microplate reader (BMG Labtech, Germany). Results were normalised to the -MMC group.

Cell viability was evaluated with calcein-AM and ethidium homodimer-1 staining. Cells were washed with HBSS and incubated with 4 μM calcein-AM (Invitrogen, ThermoFisher Scientific, Ireland) and 2 μM ethidium homodimer-1 (Invitrogen, ThermoFisher Scientific, Ireland) in HBSS for 30 min. Images were acquired with the Olympus CKX 53 microscope and processed with ImageJ.

Cell proliferation was assessed by nuclei counting. Nuclei were stained for 5 min with Hoechst 33342 (ThermoFisher Scientific, Ireland) at 1:2000 dilution in phosphate buffered saline (PBS, ThermoFisher Scientific, Ireland). Images were captured with the Olympus CKX 53 microscope and the nuclei number per field of view was automatically quantified using ImageJ. Cell counts were normalised to the -MMC group at day 4.

### 2.5 Electrophoresis analysis

Collagen type I deposition was assessed by sodium dodecyl sulphate polyacrylamide gel electrophoresis (SDS-PAGE) followed by densitometric analysis [74]. Cell layers were washed with HBSS, digested with 100 μg/ml pepsin in 0.05 M acetic acid, neutralised with 1 N NaOH, mixed with distilled water and sample buffer, denatured and loaded on 3 % stacking and 5 % separating gels. As standard (STD), bovine skin collagen type I (Symatese Biomateriaeux, France) was used, which was prepared in the same way (the loaded on the gels concentration of the STD was 500 μg/ml). Electrophoresis was performed using a mini-PROTEAN Tetra system (Bio-Rad, Ireland). The gels were stained with SilverQuest™ Silver Staining Kit (Invitrogen, ThermoFisher Scientific, Ireland). Collagen α1(I) and α2(I) bands were quantified using ImageJ and compared to the STD. Results are presented as fold-change relative to the -MMC group.

### 2.6 Immunofluorescence analysis

hTCs were cultured on Lumox® 24-well plates at 25,000 cells/cm². Cells were washed with HBSS and fixed with 2 % paraformaldehyde in PBS, blocked with 3 % bovine serum albumin in PBS for 30 min and incubated for 90 min at room temperature with mouse monoclonal antibody anti-COL I (sc-59772, Santa Cruz Biotechnology, United States) diluted 1:200 in PBS. Secondary antibody staining was performed for 30 min at room temperature with donkey anti-mouse AlexaFluor® 488 (A-21202, ThermoFisher Scientific, Ireland) diluted 1:400 in PBS. Nuclei were counterstained with Hoechst 33342 diluted 1:2000 in PBS. Fluorescence images were captured with the Olympus CKX 53 microscope and quantified with ImageJ. Data are presented as fold change difference relative to the -MMC group.

### 2.7 Correlation between physicochemical properties and collagen deposition

To examine the correlation between each of the physicochemical properties of the MMC agents and collagen type I deposition via electrophoresis and immunofluorescence, a linear regression model was fitted, from which the coefficient of determination (R²) was obtained. For the hydrodynamic radius, PDI and zeta potential, the values of the different MMC agents were plotted against the values of the deposited collagen type I. For the viscosity, the values of the -MMC and the values of the different MMC agents were plotted against the values of the deposited collagen type I. Correlation strength was categorised as negligible (0-0.09), weak (0.10-0.39), moderate (0.40-0.69), strong (0.70-0.89) and very strong (0.90-1.00) [75].

### 2.8 Gene expression analysis

At day 8, cells were lysed in 1 ml TRI Reagent® per well. RNA was isolated according to the manufacturer’s instructions and dissolved in 20 μl diethylpyrocarbonate-treated water. RNA concentration and purity were assessed using a DeNovix DS-11 spectrophotometer (DeNovix Inc., United States). cDNA was synthesised from 500 ng RNA per sample using a High-Capacity cDNA Reverse Transcription kit (ThermoFisher Scientific, Ireland), as per manufacturer protocol. qRT-PCR was performed on a QuantStudio™ 7 Flex system (ThermoFisher Scientific, Ireland) for 40 cycles. Target genes included tenogenic markers [scleraxis (SCX), mohawk homeobox (MKX), collagen type I (COL1A1), collagen type III (COL3A1), decorin (DCN), tenomodulin (TNMD), tenascin-C (TNC)], osteogenic markers [runt-related transcription factor 2 (RUNX2), collagen type X (COL10A1), osteocalcin (pmf1BGLAP), integrin binding sialoprotein (IBSP)], chondrogenic markers [SRY-box transcription factor 9 (SOX9), collagen type II (COL2A1), aggrecan (ACAN), cartilage oligomeric matrix protein (COMP)] and ageing, senescence and inflammation markers [apolipoprotein D (APOD), P16^INK4A^, cyclin D1 (CCND1) and interleukin 6 (IL6), respectively]. Glyceraldehyde-3-phosphate dehydrogenase (GAPDH) was used as reference gene. Primer IDs can be found in **Table S1**. Data analysis was performed using the 2^-ΔΔCt^ method [76] with the -MMC group as control. Fold changes > 2 or < 0.5 were considered significant.

### 2.9 Statistical analysis

Results are presented as mean ± standard deviation. Equal variance was assessed with the Brown-Forsythe test and normality was assessed with the Shapiro-Wilk test. If equal variance and/or normal distribution were not confirmed, non-parametric Kruskal-Wallis with Dunn’s multiple comparisons testing was used. Otherwise, one-way ANOVA with Holm-Sidak correction was applied. *p* < 0.05 was considered significant. All analyses were conducted using GraphPad Prism 9 (Version 9.5.1, GraphPad Software, United States).

## 3 Results

### 3.1 Physicochemical analysis

**Fig. 1:**
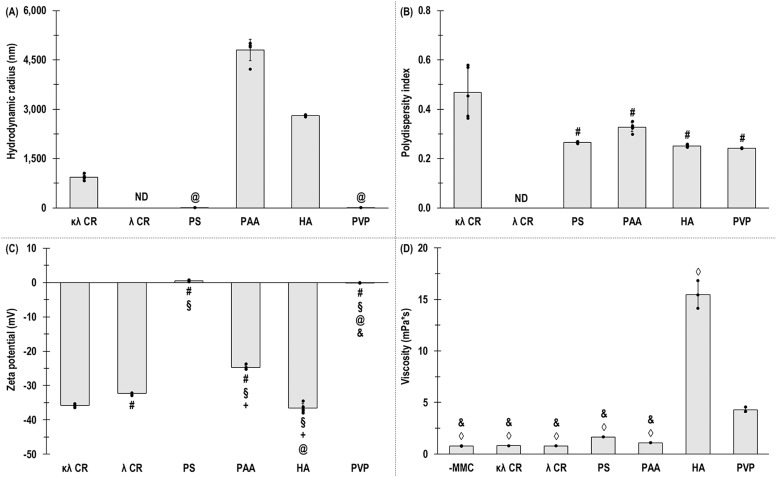
Hydrodynamic radius (A), polydispersity index (B), zeta potential (C) and viscosity (D) of κλ CR, λ CR, PS, PAA, HA and PVP. ND: not detected. @: significant (*p* < 0.05) difference compared to PAA. #: significant (*p* < 0.05) difference compared to κλ CR. §: significant (*p* < 0.05) difference compared to λ CR. +: significant (*p* < 0.05) difference compared to PS. &: significant (*p* < 0.05) difference compared to HA. ◊: significant (*p* < 0.05) difference compared to PVP. *N* = 5 for hydrodynamic radius, polydispersity index and zeta potential. N = 3 for viscosity.

PAA exhibited significantly (*p* < 0.05) higher hydrodynamic radius than PS and PVP (Fig. 1A). κλ CR had the highest (*p* < 0.05) PDI (Fig. 1B) and a relatively broad particle size distribution (**Fig. S1**). κλ CR and HA had significantly (*p* < 0.05) higher negative charge than the other MMC agents and PAA had significantly (*p* < 0.05) higher negative charge than PS and PVP (Fig. 1**C**). HA exhibited the highest (p < 0.05) viscosity (Fig. 1**D**).

### 3.2 Basic cell function analysis

No differences in cell morphology (**Fig. S2A**) and viability (**Fig. S2B**) were observed between the groups at any time point. With respect to metabolic activity (**Fig. S3A**), at day 4, HA and PVP showed a significant (*p* < 0.05) reduction compared to -MMC; at day 6, no significant (p < 0.05) differences were observed between groups; and at day 8, κλ CR and PAA showed a significant (*p* < 0.05) reduction compared to -MMC. With regards to cell proliferation (**Fig. S3B**), λ CR had the highest (*p* < 0.05) cell proliferation at every time point and at day 6 and day 8, κλ CR and PAA had significantly (*p* < 0.05) lower cell proliferation than -MMC.

### 3.3 Collagen deposition analysis and correlation to physicochemical properties

**Fig. 2:**
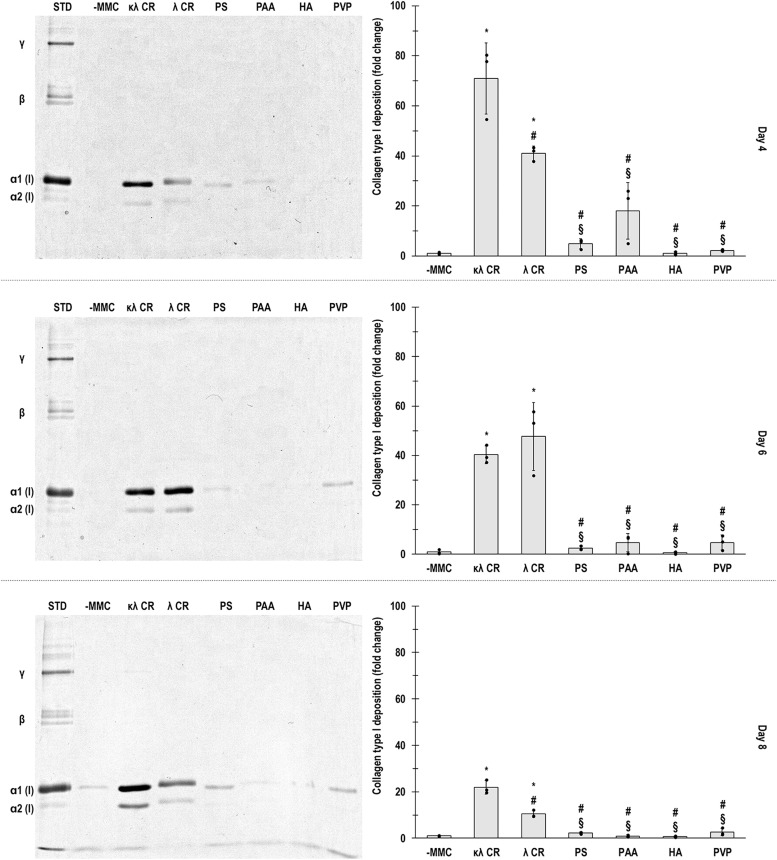
hTC collagen type I deposition, evaluated by SDS-PAGE gels (left) and respective densitometric analysis expressed as fold change over the -MMC group (right), without (-MMC) and with various MMC agents (κλ CR, λ CR, PS, PAA, HA, PVP) after 4, 6 and 8 days in culture. *: significant (p < 0.05) difference compared to -MMC. #: significant (*p* < 0.05) difference compared to κλ CR. §: significant (*p* < 0.05) difference compared to λ CR. *N* = 3.

**Fig. 3:**
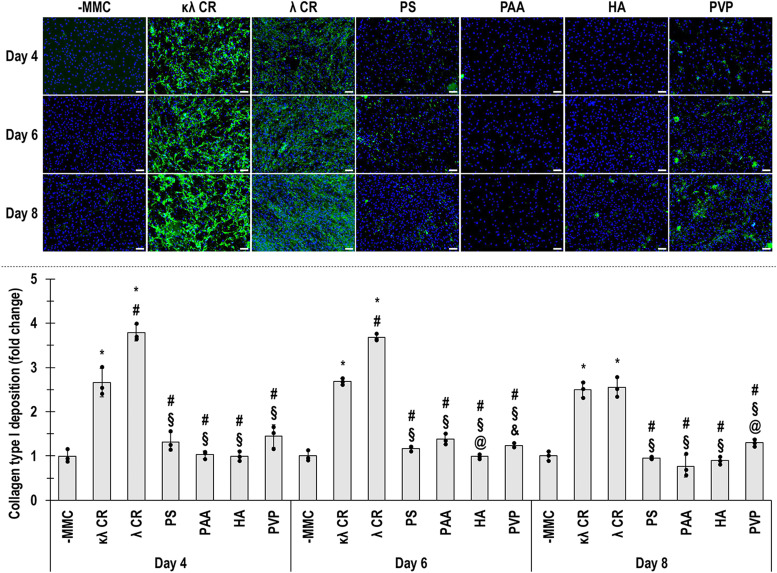
hTC collagen type I deposition evaluated by immunofluorescence (up) and respective fluorescence intensity analysis expressed as fold change over the -MMC group (down) without (-MMC) and with various MMC agents (κλ CR, λ CR, PS, PAA, HA, PVP) after 4, 6 and 8 days in culture. *: significant (*p* < 0.05) difference compared to -MMC. #: significant (*p* < 0.05) difference compared to κλ CR. §: significant (*p* < 0.05) difference compared to λ CR. @: significant (*p* < 0.05) difference compared to PAA. &: significant (*p* < 0.05) difference compared to HA. Green: collagen type I. Blue: nuclei. Scale bars: 100 μm. *N* = 3.

SDS-PAGE and densitometry analyses (Fig. 2) and immunofluorescence and fluorescence intensity analyses (Fig. 3) revealed that at day 4, day 6 and day 8, only κλ CR and λ CR induced significantly (*p* < 0.05) higher to -MMC COL I deposition. Fitting a linear regression model between collagen deposition and each physiochemical property (**Fig. S4** via immunofluorescence and **Fig. S5** for electrophoresis) revealed that only PDI induced R^2^ values ranging from moderate (0.68 at day 8 via immunofluorescence) to very strong (0.98 at day 4 via electrophoresis).

### 3.4 Gene expression analysis

**Fig. 4:**
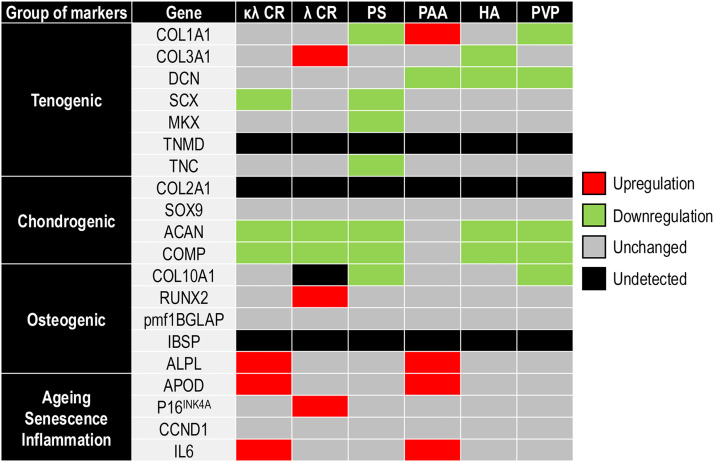
hTC gene expression heatmap of tenogenic (COL1A1, COL3A1, DCN, SCX, MKX, TNMD, TNC), chondrogenic (COL2A1, SOX9, ACAN, COMP), osteogenic (COL10A1, RUNX2, pmf1BGLAP, IBSP, ALPL) and ageing, senescence and inflammation (APOD, p16^INK4A^, CCND1, IL6) markers without (-MMC) and with various MMC agents (κλ CR, λ CR, PS, PAA, HA, PVP) after 8 days in culture. *N* = 3.

Gene expression analysis (Fig. 4 and **Fig. S6** to **Fig. S9**) revealed that κλ CR significantly (p < 0.05) downregulated SCX, ACAN and COMP and upregulated ALPL, APOD and IL6. λ CR significantly (*p* < 0.05) downregulated ACAN and COMP and upregulated COL3A1, RUNX2 and P16^INK4A^. PS significantly (p < 0.05) downregulated COL1A1, SCX, MKX, TNC, ACAN, COMP and COL10A1. PAA significantly (p < 0.05) downregulated DCN and upregulated COL1A1, ALPL, APOD and IL6. HA significantly (p < 0.05) downregulated COL3A1, DCN, ACAN and COMP. PVP significantly (p < 0.05) downregulated COL1A1, DCN, ACAN, COMP and COL10A1. TNMD, COL2A1 and IBSP were not detected in any condition.

## 4 Discussion

Despite significant advances in tendon engineering, translation to the clinic remains hindered by long *in vitro* culture times and loss of TC phenotype [73]. MMC can shorten these culture periods, but the optimal crowder for tendon applications remains undefined. Here, we evaluated six MMC agents in hTC culture, focusing on COL I deposition, cell function and gene expression and linked COL I deposition to physicochemical properties of the MMC agents.

### 4.1 Physical analysis

κλ CR had the highest PDI; HA and PAA carried the strongest negative charge; and HA and PVP increased viscosity. These results align with previous studies showing that high molecular weight, presence of negatively charged functional groups (e.g. sulphate for CR and carboxylic for PAA and HA) or hydrophilicity can influence charge, hydrodynamic radius and viscosity [[77], [78], [79], [80], [81], [82], [83], [84]]. PDI values for κλ CR were consistent with the naturally broad distribution of CR [70,72]. Only HA and PVP increased solution viscosity. The high molecular weight and the semi-flexible chain of HA give rise to highly viscus solutions with non-Newtonian flow properties [85]. With respect to PVP, a previous study has shown a solution viscosity increase with increasing molecular weight [86], although the addition of inorganic salts has been shown to decrease intrinsic viscosity [87].

### 4.2 Basic cell function analysis

None of the MMC agents caused changes in cell morphology or viability. λ CR consistently enhanced proliferation, in agreement with previous studies on mesenchymal stromal cells [67]. κλ CR and PAA reduced proliferation, likely due to higher concentration (κλ CR) or medium acidification (PAA) [88,89]. However, these effects were not accompanied by increased cell death or a sustained decrease in metabolic activity and both CR and PAA have shown acceptable safety in preclinical models [[90], [91], [92]]. Thus, these reductions are unlikely to be biologically significant.

### 4.3 Electrophoresis and immunofluorescence analysis

Electrophoresis and immunofluorescence showed that κλ CR and λ CR strongly increased collagen type I deposition at all time points, confirming previous reports that CR is the most effective MMC agent for collagen deposition across cell types [88,93]. PS, HA and PVP, although effective in other cell types [94,95], did not enhance ECM deposition in hTC cultures, in line with prior observations [60,72,83]. Correlation analysis identified PDI as the parameter most strongly associated with collagen accumulation, while charge, hydrodynamic radius and viscosity showed weak or negligible correlations. These findings reinforce earlier work suggesting that polydispersity, rather than viscosity, underlies the efficacy of MMC agents in promoting ECM deposition [72,83,96]. Previous work on MMC has shown that high PDI enhances excluded volume effects by creating a more efficient molecular packing environment, which facilitates protein folding and assembly [97,98]. Negative charge of crowders, viscosity and hydrodynamic radius modulate diffusion and molecular collision rates but appear less influential in hTC cultures [83,[99], [100], [101], [102]]. The unique combination of high PDI and strong negative charge in CR may therefore explain its superior performance. Further, similarly to our observations, viscosity has been shown to have negligible effect on drug diffusion rates [86].

### 4.4 Gene expression analysis

A MMC agent dependent gene expression was observed, suggesting either a direct (e.g. the chemistry of the MMC agent) or an indirect (e.g. change to the microenvironment) effect of the MMC agent on cell response. Evidently, these data contradict traditional biophysical studies that possibly due to simplicity considered various MMC agents as inert macromolecules [[103], [104], [105]]. Nonetheless, our observations are in agreement with previous studies in eukaryotic cell cultures, where a MMC agent dependent cell response was observed [49,72,106]. Specifically to the work in hand, κλ CR downregulated SCX, ACAN and COMP and upregulated ALPL, APOD and IL6, which in general we consider as positive outcome. Indeed, the enhanced collagen deposition and the downregulation of SCX, an early tenogenic marker [107,108], suggest that the cells entered a more mature state. The upregulated APOD expression also ties well with the more mature state of the cells, considering that it has been detected in mature dermal fibroblasts [109]. The downregulation of traditional chondrogenic markers, such as ACAN [110] and COMP [111], was welcome as it suggests that the cells did not trans-differentiate. However, one should note that previous studies have shown no difference in ACAN expression in TC cultures from young, middle age and ovariectomised rats [112] and have shown a reduced ACAN expression of hTCs in culture as opposed to native tendon [113]. Further, hTCs in monolayer and fibrous scaffolds exhibited a downregulated expression of COMP in comparison to native tendon, but the COMP expression was upregulated in high density hTC cultures [113]. Lastly, in microcarrier hTC cultures, ACAN and COMP expression were downregulated in comparison to monolayer hTC cultures [114]. In light of these studies, this ACAN and COMP downregulation is neither positive nor negative. The upregulation of ALPL may be attributed either to the direct effect of the molecule or to the change of the microenvironment (or both). To substantiate these, one should consider that sulphated polysaccharides, including CR, have been shown to increase osteogenesis [[115], [116], [117]] and ALPL activity has been shown to increase with stiffness [118], which in our case induced by the enhanced collagen deposition. Of course, we recognise that alkaline phosphatase activity has been associated with hTCs differentiating into hypertrophic chondrocyte-like cells, but in contrast to this study, the expression of COL10A1 was unchanged in our work [119]. With respect to IL6, in equine TC cultures, exogenous IL6 supplementation resulted in upregulated expression of tenogenic (COL1A1, COL3A1) and chondrogenic (COL2A1, ACAN, COMP) genes [120], which was not observed here for any of these genes and therefore does not substantiate the notion of inflammation. We believe that this increased expression of IL6 is due to the injury, as increased IL6 expression is associated with ruptured Achilles tendon [121].

It is interesting to note that a CR subtype-dependent gene expression was observed. The reader should note that this is not surprising as commercially available CR preparations have been shown to have a broad variations in structure, composition and functionality [122]. In human umbilical cord mesenchymal stromal cell cultures, the λ CR has been shown to outperform the κλ CR in collagen deposition (when both at 10 μg/ml concentration) [67]. Here though, the opposite effect was observed and was also accompanied with increased COL3A1 gene expression. Increased COL3A1 is associated with early healing [123], which also fits well with our theory that κλ CR induced a more mature phenotype. The increased p16^INK4A^ expression can be tied to the increased proliferation that the cells exhibited when treated with the λ CR and aligns with other publications [124,125], although we admit it is surprising, as its upregulation is associated with natural aging, aging pathologies and senescence [126].

None of the other macromolecules induced a MMC effect, as judged by collagen type I deposition, and therefore discussing in detail their effect on gene expression is considered unnecessary. In brief, PS yielded data contradictory to the literature, as the downregulation of tendon genes is indicative of loss of tendon phenotype, although it has been shown to maintain corneal cell phenotype [127] and although downregulation of cartilage genes was observed, previous studies have shown to increase chondrogenic differentiation [128]. We attribute these contradictions to the no effect in collagen deposition in this work and the enhanced collagen deposition in the other studies. PAA upregulated COL1A1, ALPL, APOD and IL6 and downregulated DCN, which collectively may suggest an early phase tenogenesis, especially when at times higher, but not always significant, than the -MMC group collagen deposition was detected. The ALPL, APOD and IL6 upregulated expression can be justified in similar manner to κλ CR, whilst the upregulation of COL1A1 is associated with middle stage tendon healing, where COL1A1 increases and COL3A1 does not show any significant variation, as evidenced by previous work on the gene expression profile of damaged human Achilles tendons [129]. The downregulation of DCN (also observed in the HA and PVP groups) was welcome as its downregulation is associated with scarless wound healing and absence of fibrotic tissue [130]. The downregulation of COL3A1 by HA here observed has been previously reported in hTCs [131] and indicated favourable middle stage tendon healing, as excessive COL3A1 expression is associated with tendon adhesions [132]. PVP showed a downregulation of COL1A1, in agreement with previous work on dermal fibroblasts [71], although the lack of COL I deposition herein observed contrasts with the literature [83], suggesting once more the necessity of the optimisation of MMC agents for different cell types. Both HA and PVP downregulated chondrogenic markers, in contrast to previous observations, where both HA [133,134] and PVP [135] were shown to enhance chondrogenesis.

### 4.5 Limitations

Although our data, overall, align very well with previous publications, it is important to mention some limitations that could potentially inform future investigations. First of all, this study was conducted with cells from a young female donor and the cells were extracted via the migration protocol from one small Achilles tendon sample. The reader should note that differences in human hamstring tendon TCs [136], human semitendinosus muscle tendon TCs [137], rat Achilles tendon TCs [138] and equine superficial digital flexor tendon TCs [139] have been reported as a function of age. Differences have also been reported as a function of sex in mouse flexor digitorum longus tendon TCs [140] and mouse plantar flexor tendon TCs, albeit very subtle [141]. Despite all these, a study with human TCs from 149 patients showed no differences as a function of age (< 35, 35-44, 45-54, > 55 years old), sex (male, female) and tendon tissue (patella tendon, palmaris longus tendon) [142]. It is also worth noting that both TCs (migration protocol [143] or collagenase digestion protocol [144]) and tendon stem/progenitor cells (migration protocol [145] or collagenase digestion protocol [146]) are isolated in the same way. It should be noted that a direct comparison between these two protocols did not reveal any substantial differences, other than that the enzymatic digestion is shorter and yields remarkably higher cell number [147], possibly a highly mixed / heterogeneous population of cells. The reader should also note that FBS as opposed to more human like media was used, as in previous work we found FBS to outperform allogeneic sera in hTC cultures [60], although in equine TC cultures, allogeneic sera outperformed FBS [148]. Lastly, this work was conducted for up to 8 days in culture. As discussed, the only way that we can make healthcare provider affordable medicines is if they can be fabricated rapidly. From electrophoresis analysis, for example, it is evidenced that at the early time points, we have the highest collagen deposition (∼ 70 fold increase at day 4, ∼ 40 fold increase at day 6 and ∼ 20 fold increase at day 8 in collagen type I deposition); the application therefore of MMC pre-empts the use of prolonged cultures for the development of tissue engineered medicines.

## 5 Conclusions

Autologous hTC injection has resulted in pain reduction and improved functional outcomes in elbow [15,16] and gluteal [22] tendon pathologies and even in rotator cuff [17] and shoulder [18] pathologies of elite athletes. Upon this success stories, several clinics (e.g. https://orthocell.com; https://gcsportsmed.com.au/autologous-tenocyte-implantation/; https://melbourneradiology.com.au; https://markhamlin.com.au; https://www.drmoses.com.au) in Australia (this intervention was approved by the Australian Therapeutic Goods Administration in 2010) offer this service as single injection or multiple injections or in combination with other treatments (e.g. platelet rich plasma). However, such interventions are only suitable for tendon regeneration in cases of tendinopathy and small (< 1 cm) to medium (1-3 cm) tears, whilst in the case of large (3-5 cm) to massive (> 5 cm) full thickness tears, graft intervention is necessary. Unfortunately, although grafting approaches result in superior to repairs without grafts tendon healing and clinical outcomes, the general consensus is that further research and development work is required to develop therapies that will yield healing without fibrosis, neotissues with low re-tear rates and ultimately improved patient therapeutic outcomes [[149], [150], [151]]. In this regard, tissue engineering therapies are becoming more and more prevalent [152], including tendon engineering [153]. Regretfully, no tendon engineered product based on hTCs (or any other cell population) is currently available, largely due to the rapid hTC loss of functionality *in vitro* and the very long *in vitro* time required to manufacture one that would make it too expensive for any healthcare provider to afford. Although MMC has been shown to enhance and accelerate ECM deposition, the optimal MMC agent in hTC cultures is still elusive. Here, based on basic cell function, collagen type I deposition and gene expression analyses, we identified the κλ CR and λ CR as the most appropriate MMC agents for the development of tendon engineered substitutes, in a scaffold or scaffold-free conformation, subject to the dimensionality of the tear. Further, this work puts forward an experimental setup for identifying an appropriate MMC agent in permanently differentiated cell culture.

## Funding

This publication has emanated from research conducted with the financial support of Taighde Éireann – Research Ireland under Grant Number 19/FFP/6982. This publication is also part of a project that received funding from the European Research Council (ERC) Consolidator Grant under the European Union’s Horizon 2020 research and innovation programme under Grant Number 866126. This publication is also part of a project that received funding from the European Research Council (ERC) Proof of Concept Grant under the European Union’s Horizon Europe research and innovation programme under the Grant Number 101138593.

## Generative artificial intelligence statement

Generative artificial intelligence was not used anywhere in this study.

## CRediT authorship contribution statement

**Andrea Rossoni:** Writing – original draft, Investigation, Formal analysis. **Giovanni Lauretta:** Writing – review & editing, Investigation, Formal analysis. **Stephen Kearns:** Writing – review & editing, Resources, Methodology. **Dimitrios I. Zeugolis:** Writing – review & editing, Writing – original draft, Visualization, Validation, Supervision, Resources, Project administration, Methodology, Investigation, Funding acquisition, Formal analysis, Conceptualization.

## Declaration of competing interest

The authors declare the following financial interests/personal relationships which may be considered as potential competing interests:

Given their role as Editor-in-Chief, Dimitrios I. Zeugolis had no involvement in the peer review of this article and had no access to information regarding its peer review. Full responsibility for the editorial process for this article was delegated to another journal editor. If there are other authors, they declare that they have no known competing financial interests or personal relationships that could have appeared to influence the work reported in this paper.

## Data Availability

Data will be made available on request.

## Appendix Supplementary materials

Image, application 1
