# Supplementary material for: Comparative analysis of different macromolecular crowding agents in human tenocyte cultures
Source: Biomater Biosyst. 2026 Jan 10;21:100127. doi: 10.1016/j.bbiosy.2026.100127 (PMC12828578; doi:10.1016/j.bbiosy.2026.100127)

**Supplementary Information**

# Title

Comparative analysis of different macromolecular crowding agents in human tenocyte cultures

# Authors

Andrea Rossoni (1), Giovanni Lauretta (1), Stephen Kearns (2), Dimitrios I. Zeugolis* (1)

# Affiliations

(1) Regenerative, Modular & Developmental Engineering Laboratory (REMODEL), Charles Institute of Dermatology, Conway Institute of Biomolecular & Biomedical Research and School of Mechanical & Materials Engineering, University College Dublin (UCD), Dublin, Ireland

(2) Bon Secours Hospital, Merlin Park Regional Hospital, University Hospital Galway and University of Galway, Galway, Ireland

* Corresponding Author: Dimitrios I. Zeugolis, REMODEL, UCD. Telephone: +353 (0) 1716 1887. Email: dimitrios.zevgolis@ucd.ie

**Table S1:** Genes assessed along with their respective abbreviation and assay ID.

| **Group of markers** | **Gene name** | **Gene abbreviation** | **Assay ID** |
| --- | --- | --- | --- |
| Reference gene | Glyceraldehyde-3-phosphate dehydrogenase | GAPDH | Hs99999905_m1 |
| Tenogenic | Collagen type I alpha 1 chain | COL1A1 | Hs00164004_m1 |
|  | Collagen type III alpha 1 chain | COL3A1 | Hs00943809_m1 |
|  | Decorin | DCN | Hs01075781_m1 |
|  | Scleraxis | SCX | Hs03054634_g1 |
|  | Homeobox protein Mohawk | MKX | Hs00543190_m1 |
|  | Tenomodulin | TNMD | Hs00223332_m1 |
|  | Tenascin C | TNC | Hs01115665_m1 |
| Chondrogenic | Collagen type II alpha 1 chain | COL2A1 | Hs00264051_m1 |
|  | SRY-box transcription factor 9 | SOX9 | Hs00165814_m1 |
|  | Aggrecan | ACAN | Hs00153936_m1 |
|  | Cartilage oligomeric matrix protein | COMP | Hs00164359_m1 |
| Osteogenic | Collagen type X alpha 1 chain | COL10A1 | Hs00166657_m1 |
|  | Runt-related transcription factor 2 | RUNX2 | Hs01047973_m1 |
|  | Osteocalcin | Pmf1BGLAP | Hs07290785_m1 |
|  | Integrin binding sialoprotein | IBSP | Hs00913377_m1 |
|  | Alkaline phosphatase | ALPL | Hs01029144_m1 |
| Ageing, senescence and inflammation | Apolipoprotein D | APOD | Hs00155794_m1 |
|  | p16^INK4A^ | p16^INK4A^ | Hs00923894_m1 |
|  | Cyclin D1 | CCND1 | Hs00765553_m1 |
|  | Interleukin 6 | IL6 | Hs00985639_m1 |

**Figure S1:** Particle size distribution against particle radius of κλ CR (A), λ CR (B), PS (C), PAA (D), HA (E) and PVP (F). N = 5.


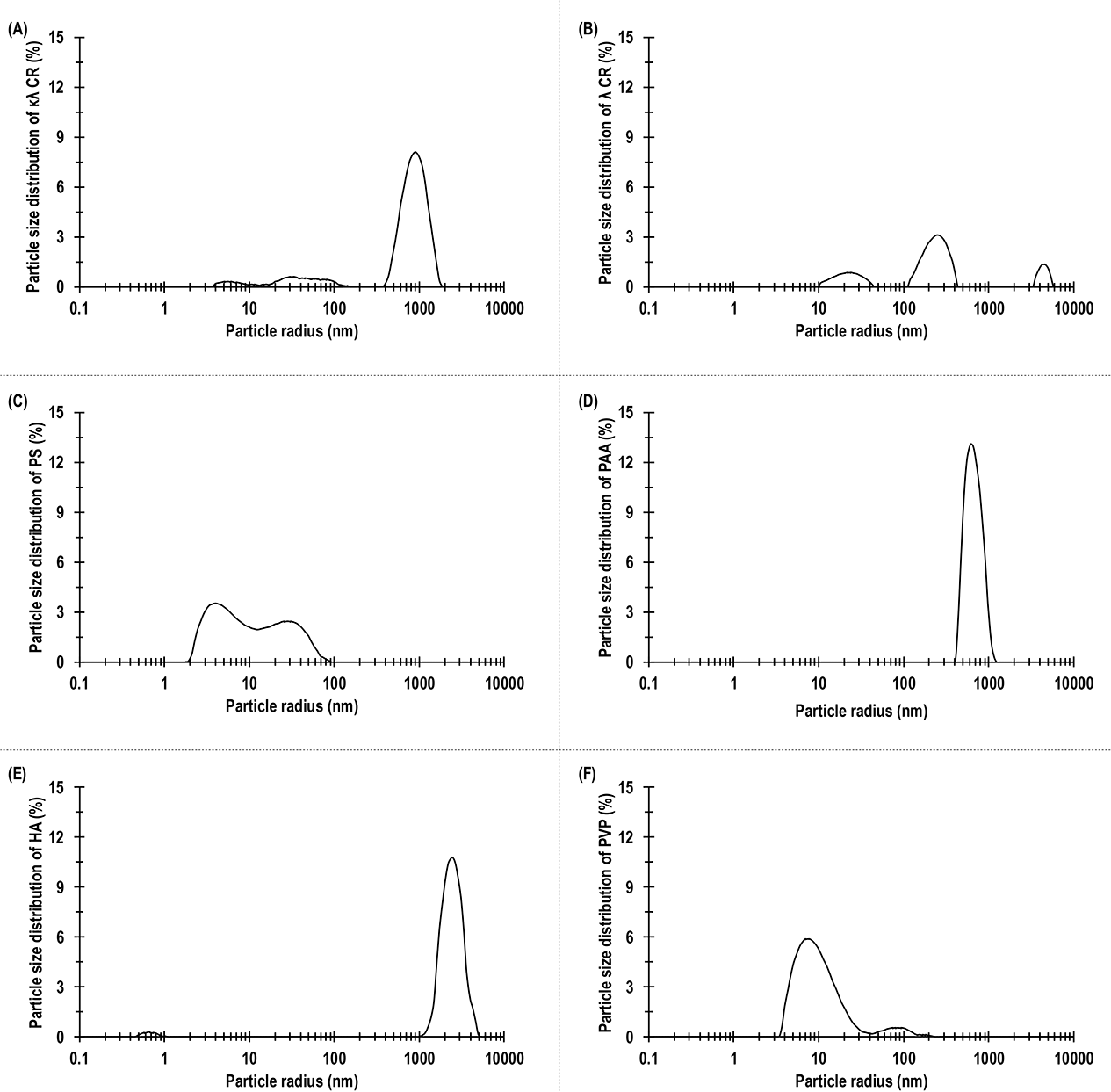


**Figure S2:** hTC morphology (A) and viability (B; green indicates live cells and red indicates dead cells) without (-MMC) and with various MMC agents (κλ CR, λ CR, PS, PAA, HA, PVP) after 4, 6 and 8 days in culture. Scale bars: 100 μm. N = 3.


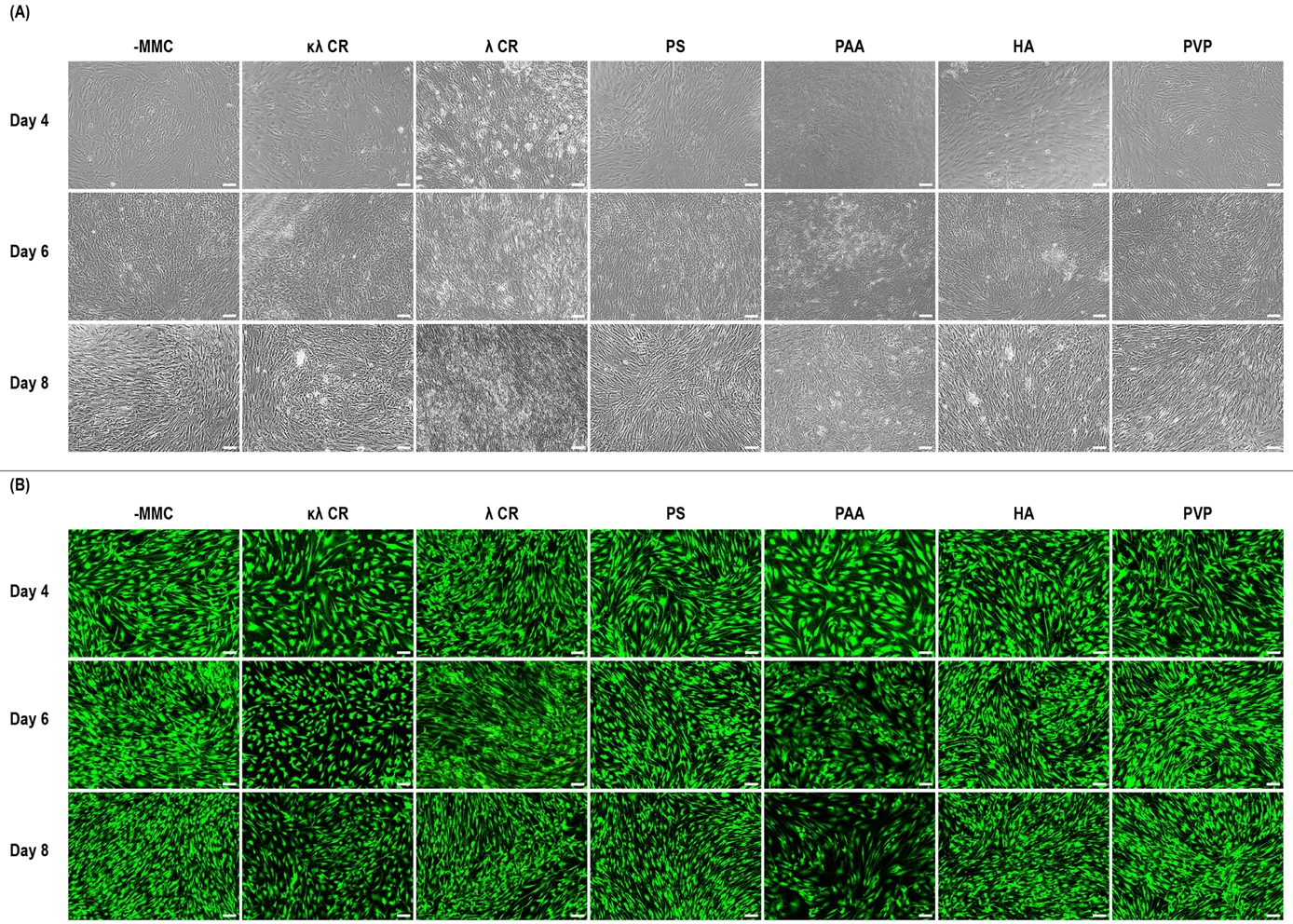


**Figure S3:** hTC metabolic activity (A) and proliferation (B) without (-MMC) and with various MMC agents (κλ CR, λ CR, PS, PAA, HA, PVP) after 4, 6 and 8 days in culture. *: significant (p < 0.05) difference compared to -MMC. §: significant (p < 0.05) difference compared to λ CR. #: significant (p < 0.05) difference compared to κλ CR. +: significant (p < 0.05) difference compared to PS. @: significant (p < 0.05) difference compared to PAA. Scale bars: 100 μm. N = 3.


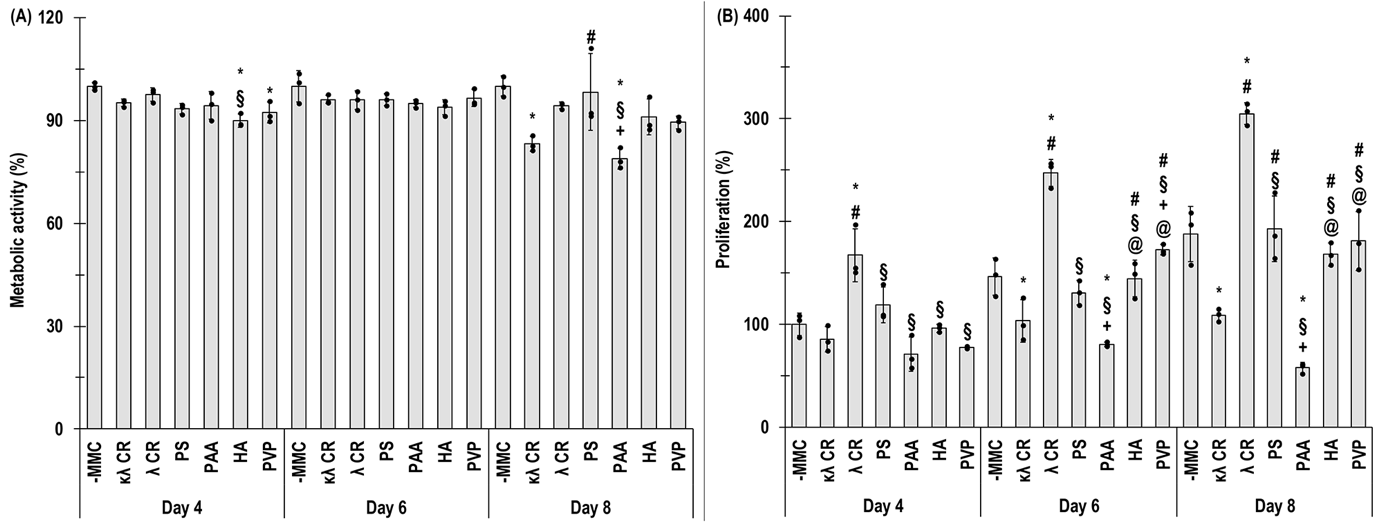


**Figure S4:** Correlation analysis of collagen type I deposition, evaluated by immunofluorescence, after 4, 6 and 8 days in hTC cultures without (-MMC) and with various MMC agents (κλ CR, λ CR, PS, PAA, HA, PVP) versus hydrodynamic radius (A), PDI (B), zeta potential (C) and viscosity (D).


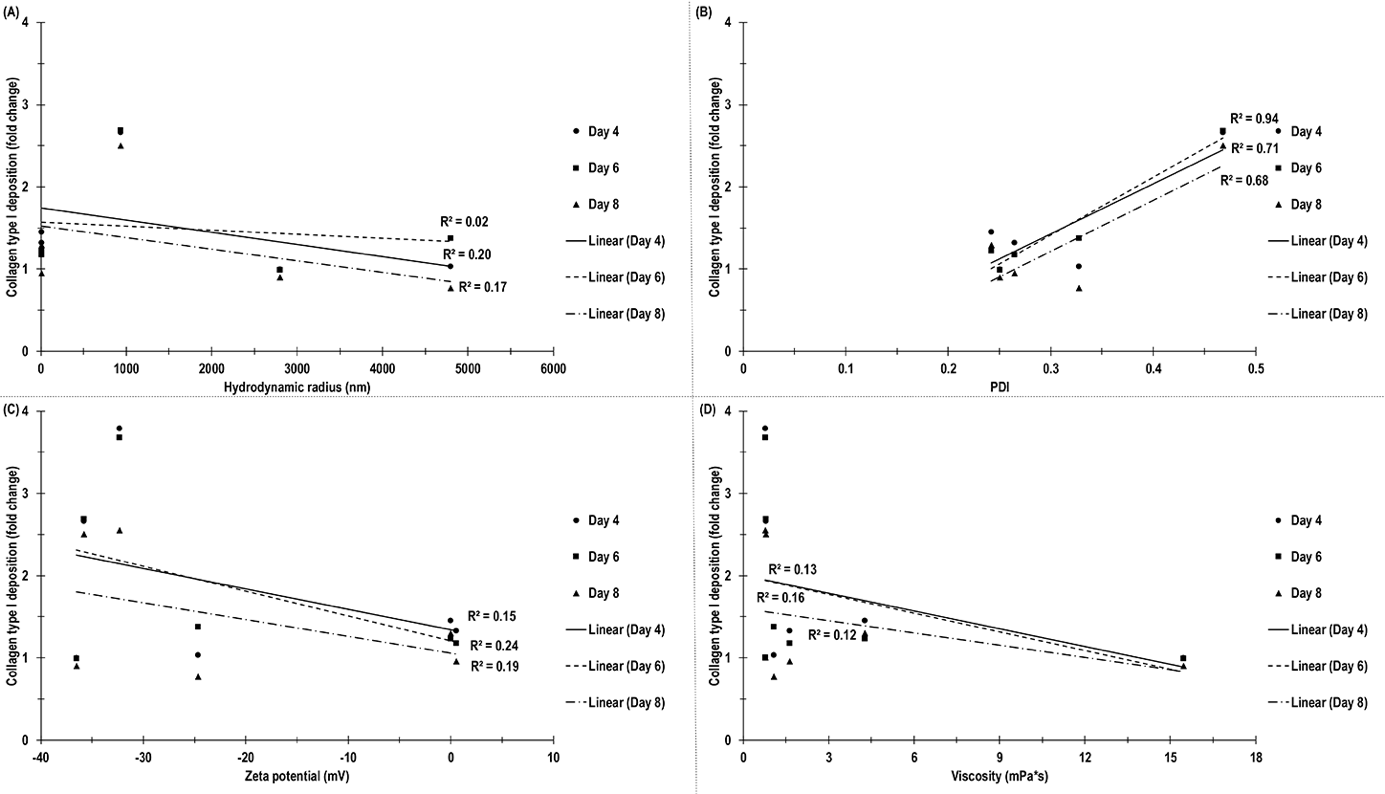


**Figure S5:** Correlation analysis of collagen type I deposition, evaluated by electrophoresis, after 4, 6 and 8 days in hTC cultures without (-MMC) and with various MMC agents (κλ CR, λ CR, PS, PAA, HA, PVP) versus hydrodynamic radius (A), PDI (B), zeta potential (C) and viscosity (D).


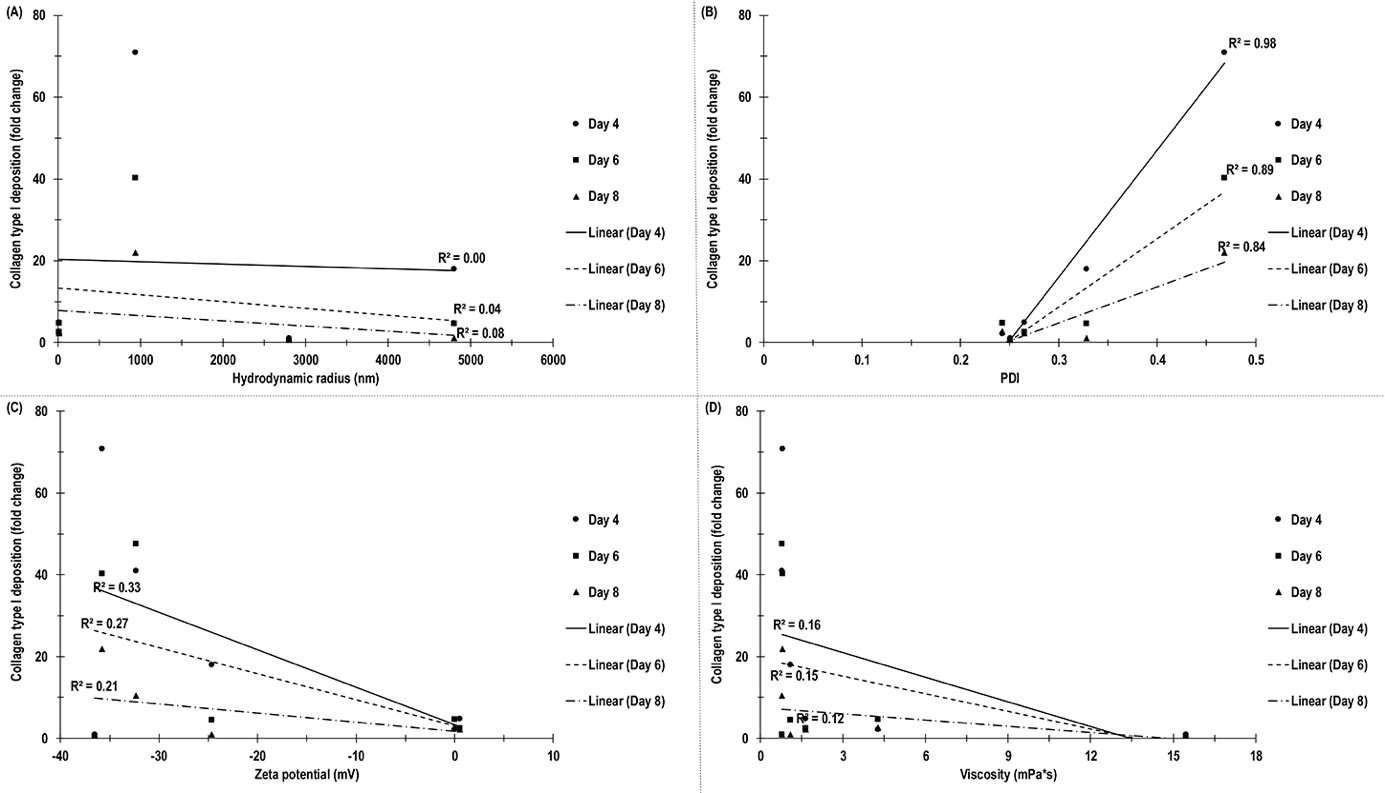


**Figure S6:** hTC gene expression fold-change plots of tenogenic markers (COL1A1, COL3A1, DCN, SCX, MKX, TNMD, TNC) without (-MMC) and with various MMC agents (κλ CR, λ CR, PS, PAA, HA, PVP) after 8 days in culture. *: significant (p < 0.05) difference compared to -MMC. N = 3.


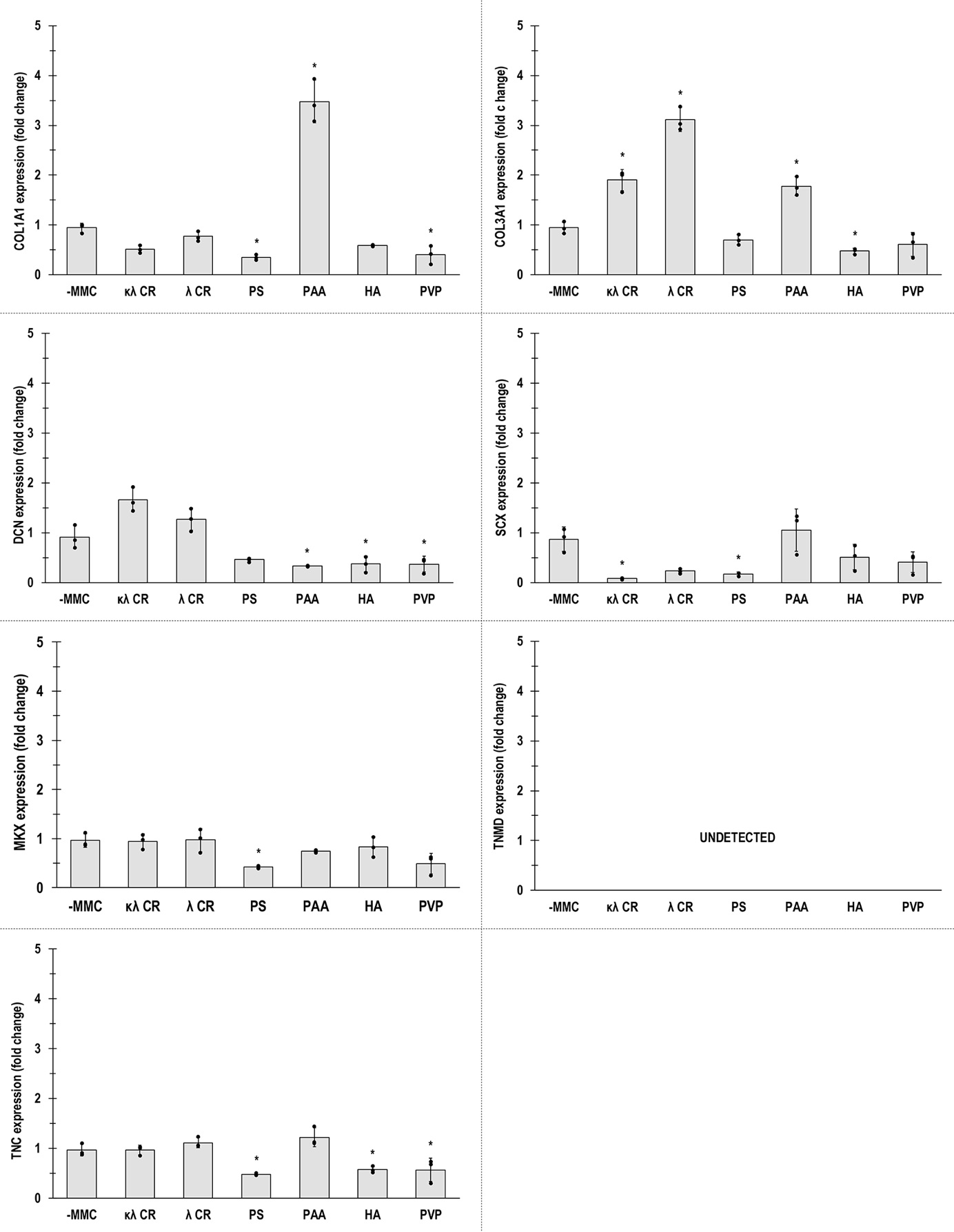


**Figure S7:** hTC gene expression fold-change plots of chondrogenic markers (COL2A1, SOX9, ACAN, COMP) without (-MMC) and with various MMC agents (κλ CR, λ CR, PS, PAA, HA, PVP) after 8 days in culture. *: significant (p < 0.05) difference compared to -MMC. N = 3.


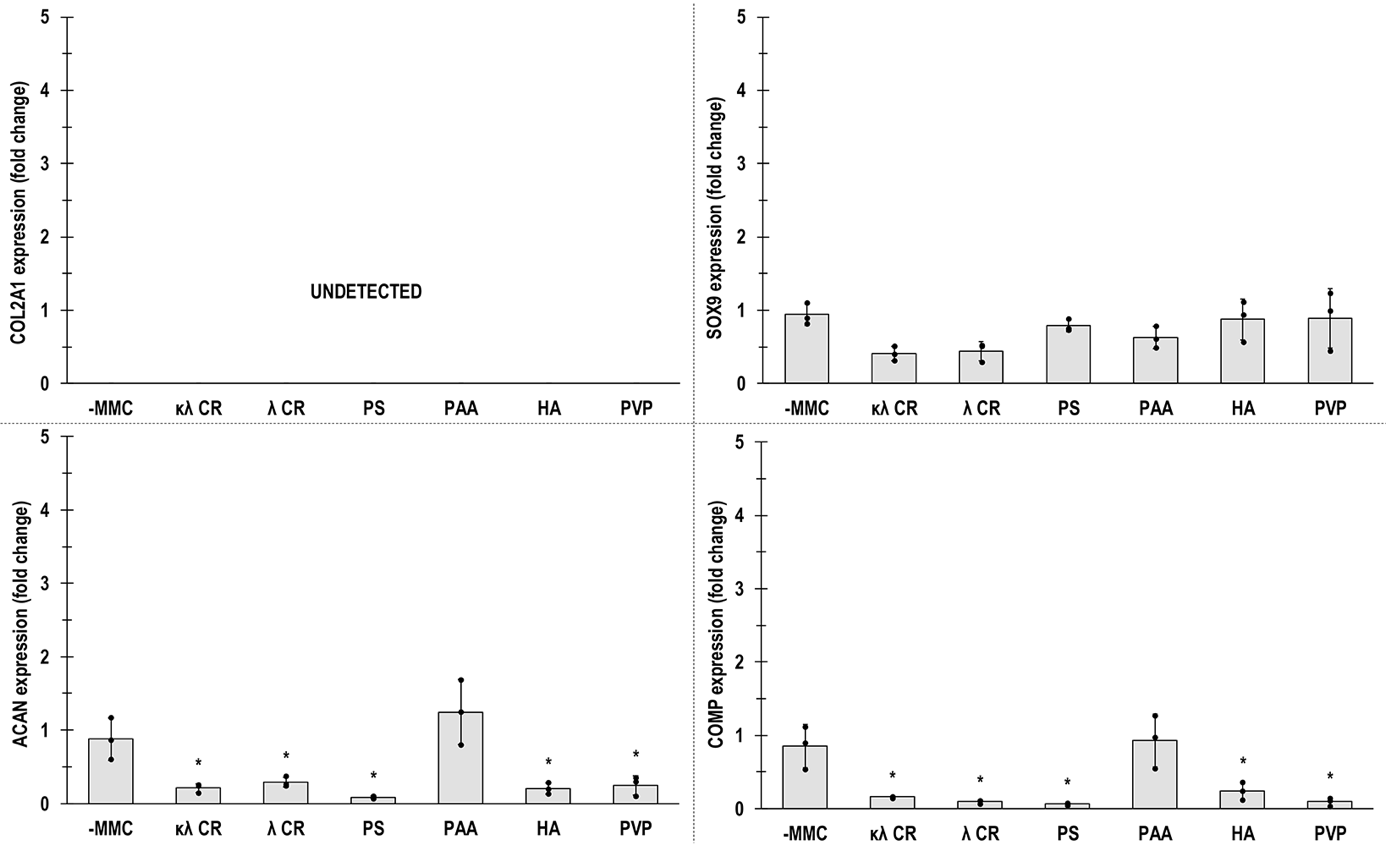


**Figure S8:** hTC gene expression fold-change plots of osteogenic markers (COL10A1, RUNX2, pmf1BGLAP, IBSP, ALPL) without (-MMC) and with various MMC agents (κλ CR, λ CR, PS, PAA, HA, PVP) after 8 days in culture. *: significant (p < 0.05) difference compared to -MMC. N = 3.


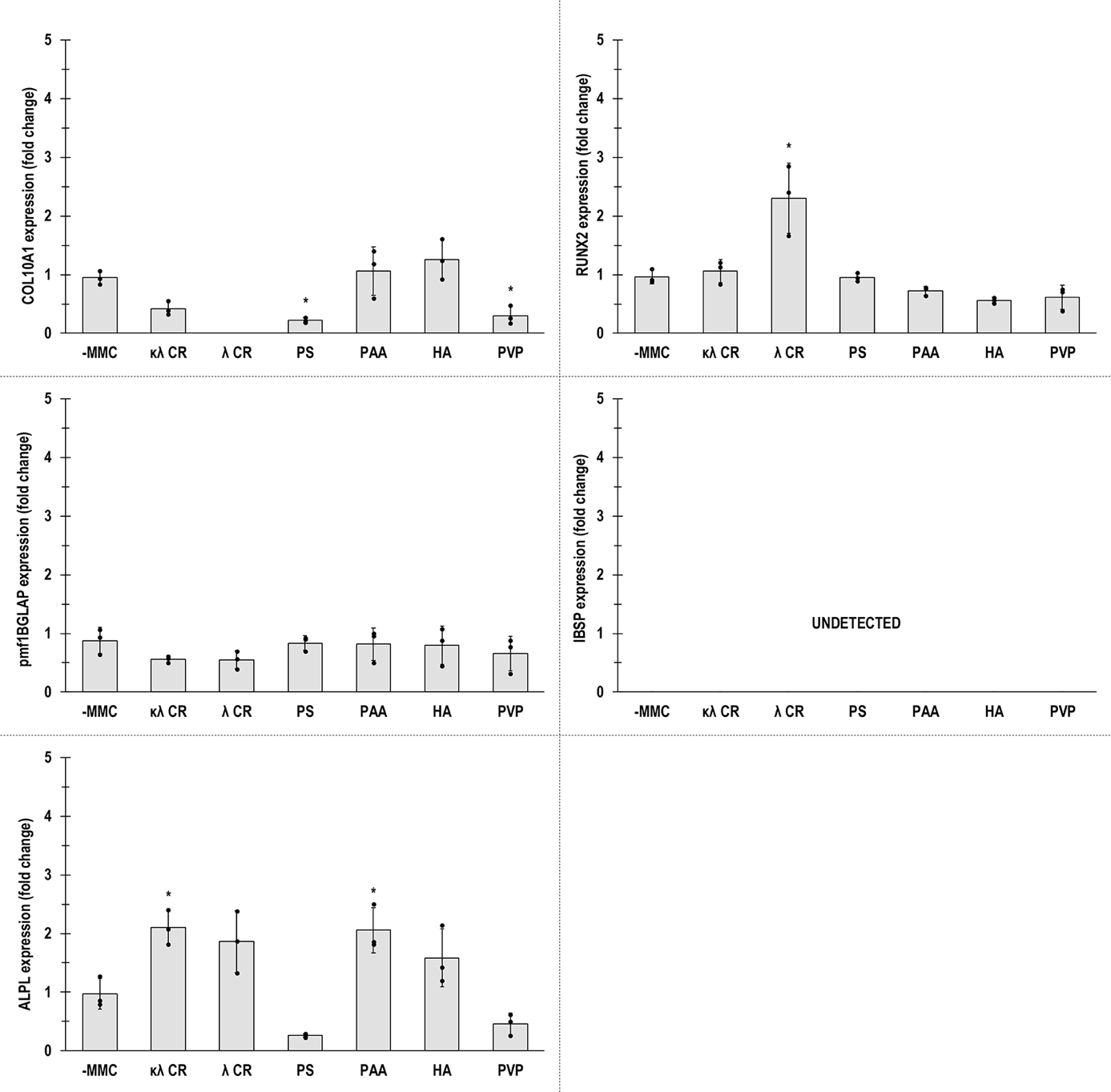


**Figure S9:** hTC gene expression fold-change plots of ageing, senescence and inflammation markers (APOD, p16^INK4A^, CCND1, IL6) without (-MMC) and with various MMC agents (κλ CR, λ CR, PS, PAA, HA, PVP) after 8 days in culture. *: significant (p < 0.05) difference compared to -MMC. N = 3.


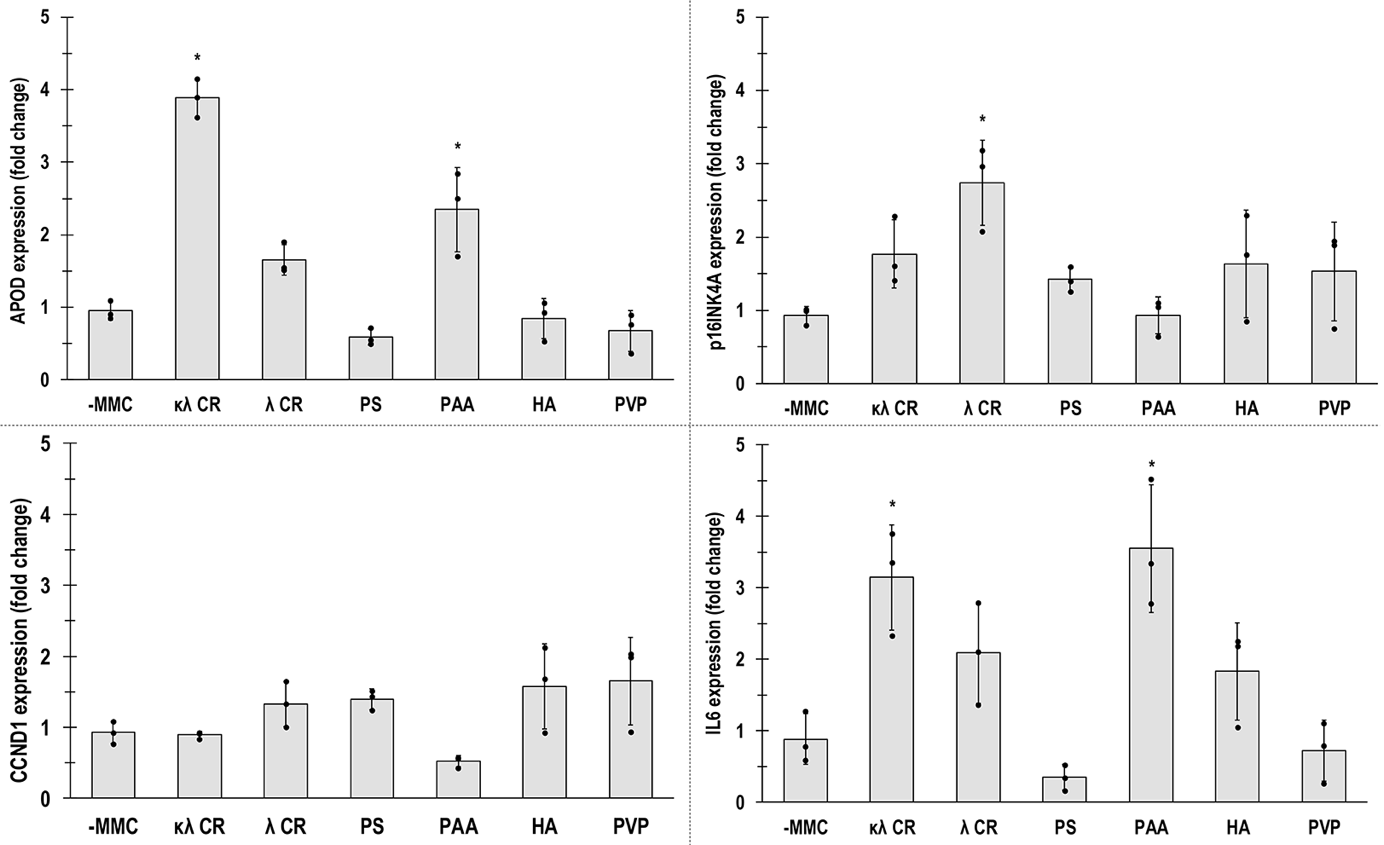

Supplement: Supplementary file 1 [file mmc1.docx]
